# Empagliflozin activates Sestrin2-mediated AMPK/mTOR pathway and ameliorates lipid accumulation in obesity-related nonalcoholic fatty liver disease

**DOI:** 10.3389/fphar.2022.944886

**Published:** 2022-09-05

**Authors:** Yuting Ma, Guangdong Zhang, Zenggguang Kuang, Qian Xu, Tongtong Ye, Xue Li, Na Qu, Fang Han, Chengxia Kan, Xiaodong Sun

**Affiliations:** ^1^ Department of Endocrinology and Metabolism, Affiliated Hospital of Weifang Medical University, Weifang, China; ^2^ Clinical Research Center, Affiliated Hospital of Weifang Medical University, Weifang, China; ^3^ Department of Pathology, Affiliated Hospital of Weifang Medical University, Weifang, China

**Keywords:** nonalcoholic fatty liver disease, free fatty acids, empagliflozin, inflammation, AMPK-mTOR

## Abstract

Empagliflozin (EMPA) therapy has led to improvements in patients with non-alcoholic fatty liver disease (NAFLD). Sestrin2 is a stress-inducible protein that controls the AMPK-mTOR pathway and inhibits oxidative damage in cells. This study investigated the functional implications of EMPA on the multifactorial pathogenesis of NAFLD and potential underlying molecular mechanisms of pathogenesis. An *in vitro* model of NAFLD was established by treating HepG2 cells with palmitic acid (PA); an *in vivo* model of NAFLD was generated by feeding C57BL/6 mice a high-fat diet. Investigations of morphology and lipid deposition in liver tissue were performed. Expression patterns of Sestrin2 and genes related to lipogenesis and inflammation were assessed by reverse transcription polymerase chain reaction. Protein levels of Sestrin2 and AMPK/mTOR pathway components were detected by Western blotting. NAFLD liver tissues and PA-stimulated HepG2 cells exhibited excessive lipid production and triglyceride secretion, along with upregulation of Sestrin2 and increased expression of lipogenesis-related genes. EMPA treatment reversed liver damage by upregulating Sestrin2 and activating the AMPK-mTOR pathway. Knockdown of Sestrin2 effectively increased lipogenesis and enhanced the mRNA expression levels of lipogenic and pro-inflammatory genes in PA-stimulated HepG2 cells; EMPA treatment did not affect these changes. Furthermore, Sestrin2 knockdown inhibited AMPK-mTOR signaling pathway activity. The upregulation of Sestrin2 after treatment with EMPA protects against lipid deposition-related metabolic disorders; it also inhibits lipogenesis and inflammation through activation of the AMPK-mTOR signaling pathway. These results suggest that Sestrin2 can be targeted by EMPA therapy to alleviate lipogenesis and inflammation in obesity-related NAFLD.

## Introduction

Non-alcoholic fatty liver disease (NAFLD) is a chronic metabolic disease characterized by excessive triglycerides accumulation without heavy alcohol consumption (Neuschwander-Tetri, 2017; Younossi, 2019). The increased prevalence of obesity worldwide is associated with an increase in global NAFLD risk. Triglycerides accumulated in liver labeling with single steatosis, followed by hepatic steatosis, steatohepatitis, cirrhosis, and even hepatocellular carcinoma (Viganò et al., 2018). Obesity-related increases in free fatty acids (FFAs) lead to enhanced lipotoxic metabolite production, thus resulting in a pro-inflammatory phenotype accompanied by increased expression of pro-inflammatory cytokines (Haukeland et al., 2006; Lucero et al., 2011; Marra and Lotersztajn, 2013). FFAs can also increase intracellular lipid accumulation, leading to hepatic lipotoxicity through its effects on the regulatory patterns of various genes (Chu et al., 2013). Additionally, FFAs impair endoplasmic reticulum function and initiate the unfolded protein response (Liu et al., 2010). This FFA-induced lipotoxicity causes lipid oxidation and eventually progresses to excessive lipid peroxidation, which is associated with enhanced risk of NAFLD pathogenesis (Zámbó et al., 2013; Mota et al., 2016).

Sodium-glucose cotransporter-2 inhibitors (SGLT-2is) are novel hypoglycemic medications used to treat diabetes (Kramer and Zinman, 2019). SGLT-2is decrease blood glucose levels by enhancing glycosuria and diuresis; these changes produce additional benefits, including body weight reduction (Kramer and Zinman, 2019). Previous clinical trials and meta-analyses have shown that SGLT-2is [e.g., empagliflozin (EMPA), canagliflozin, and dapagliflozin] have protective functions in patients with heart failure, cardiovascular diseases, and/or diabetic nephropathy (Fitchett et al., 2019; Li et al., 2021; Tuttle et al., 2021). Additionally, SGLT-2is can maintain liver structure and function in patients with NAFLD (Coelho et al., 2020; Mantovani et al., 2020). Moreover, the use of SGLT-2is has led to improvements in histologic steatosis, lobular inflammation, hepatocyte ballooning, and fibrosis in patients with liver steatosis (Hsiang and Wong, 2020). Thus, SGLT-2i therapy appears to be a promising strategy for management of NAFLD.

Sestrin2 (Sesn2) is a highly conserved stress-inducible protein expressed in multiple tissues (e.g., liver, muscles, heart, and kidneys); it acts as a critical regulator of obesity-associated pathologies (Gong et al., 2021). As a stress-inducible metabolic protein, Sesn2 participates in the maintenance of metabolic homeostasis by regulating responses to various stresses, including oxidative stress and metabolic pathologies (Sun W. et al., 2020). Consistent with this function, sestrin2 knockout mice demonstrated increased fibrosis and the enhancement of inflammatory responses, apoptosis, and reactive oxygen species production (Hwang et al., 2017). Previous studies showed that Sesn2 prevents NAFLD and protects against hepatic fibrosis induced by various drugs (Yang et al., 2019; Han et al., 2020). Increased Sesn2 expression reportedly protects against hepatic steatosis, attenuates hepatic endoplasmic reticulum stress, and alleviates fibrosis in obese mice (Park et al., 2014; Jegal et al., 2020). Moreover, Sesn2 can activate the AMPK pathway and suppress mTOR signaling to attenuate various metabolic disorders, including insulin resistance, mitochondrial dysfunction, and cardiac dysfunction (Sun X. et al., 2020; Kishimoto et al., 2021). Based on the previous literature summarized above, we hypothesized that Sesn2 could serve as a novel therapeutic target for various aging- and obesity-associated diseases.

While SGLT2is are regarded as key regulators of lipid metabolism in NAFLD therapy, the underlying mechanisms have not been fully elucidated. Here, we aimed to explore the potential mechanism by which EMPA protects against lipogenesis and inflammation through assessment of Sesn2-mediated AMPK-mTOR signaling and cellular antioxidant signaling in a model of high-fat diet (HFD)-induced NAFLD.

## Materials and methods

### Materials

A detailed list of materials is provided in Supplementary Table S1.

### Animals

Six-week-old male C57BL/6J mice (Shandong Jinan Pengyue Animal Breeding Co., Ltd.) were housed in ventilated cages (five mice per cage). All mice were randomly assigned to one of the following three groups: the normal control group was fed normal chow (10% fat, 320 kcal per 100 g); the HFD and EMPA groups were fed an HFD (54% fat, 529.8 kcal per 100 g) for 12 weeks to establish the NAFLD model. Then, the EMPA group was received EMPA (10 mg/kg/d diluted in saline) by oral gavage) for another 8 weeks, whereas the other two groups received saline. Body fat in mice was measured using the Mouse-Body Composition Analyzer (Bruker, Germany). All mice were housed under standard laboratory conditions (temperature, 22 ± 1°C; humidity, 55%–60%; 12-h/12-h light and dark cycles). All animal experiments were approved by the Animal Ethics Committee of Weifang Medical University.

### Cell culture

The HepG2 human hepatocyte cell line was grown in Dulbecco’s modified Eagle medium supplemented with 10% fetal bovine serum. All cells were maintained at 37°C in a humidified incubator. For *in vitro* experiments, cells were seeded and then cultured to a density of 70%–80%; subsequently, they were supplemented with 0.5 mM palmitic acid (PA) for 24 h, then treated with 1 μM EMPA for 24 h (Sun X. et al., 2020). Finally, the cells were collected for future analysis.

### Biochemical analyses

Mice were anesthetized with 2% sodium pentobarbital via intraperitoneal injection. Blood samples were obtained, and the liver was collected and weighed. Serum alanine aminotransferase, aspartate aminotransferase, FFA, lipid peroxidation, malondialdehyde (MDA), and triglyceride levels were determined using commercial kits. Total liver or cellular lipids were extracted; hepatic triglyceride, lipid peroxidation, and MDA contents were quantified according to the manufacturer’s instructions.

### Hematoxylin and eosin and Oil Red O staining

Liver tissues were fixed overnight with 4% paraformaldehyde. In accordance with standard protocols, the tissue sections were subjected to H&E staining and ORO staining. HepG2 cells were subjected to ORO staining using an ORO Stain Kit (Solarbio, Beijing, China). After treatment, cells were washed and then fixed with ORO Fixative. Subsequently, the cells were briefly washed and soaked in ORO reagent for 30 min, then washed with distilled water and subjected to nuclear staining with Mayer’s hematoxylin for 1 min. Finally, red-stained lipid droplets of the cells were visualized via microscopy. ORO contents were quantified by the addition of 100% isopropanol to each well and measurement of sample absorbance at 495 nm using a microplate reader.

### Cell transfection

siRNA directed against Sesn2 (siSESN2; sequences are listed in Supplementary Table S2) and negative control siRNA (siNC) were purchased from Qingke Co., Ltd. (Beijing, China). Cell transfection was performed using Lipofectamine 3000. HepG2 cells were seeded in six-well plates at 2.5×10^5^ cells/well. Lipofectamine-3000 reagent was diluted in 125 μl of OPTI-MEM (Invitrogen); siSESN2 or siNC (7.5 μl) was diluted in the same volume of OPTI-MEM in a separate tube. The diluted siRNA and Lipofectamine-3000 were then mixed and incubated for 15 min. Before subsequent experiments, the cells were cultured for 6 h; the medium was then replaced with fresh Dulbecco’s modified Eagle medium for 48 h siSESN2 knockdown efficiency was examined by reverse transcription polymerase chain reaction (RT-PCR) and Western blotting. Before harvest, the transfected cells were stimulated with 0.5 mM PA or treated with or without 1 μM EMPA for 24 h.

### Western blot analysis

Equal amounts of total protein were resolved via 10% sodium dodecyl sulfate–polyacrylamide gel electrophoresis, then transferred to polyvinylidene fluoride membranes. The membranes were incubated with anti-Sesn2, anti-Nrf2, anti-PGC1α, anti-p-mTOR, anti-p-AMPK (Thr172), anti-AMPK, anti-mTOR, or anti-HO-1 antibodies (1:1000) and anti-β-actin (1:5000) at 4°C overnight. The membranes were then incubated with the secondary antibody for 1 h. Blots were then developed by enhanced chemiluminescence and analyzed using ImageJ software (NIH, Bethesda, MD, United States).

### Quantitative PCR analysis

Total RNA was isolated from cell lines or liver tissues using TRIzol. RNA was reverse transcribed to cDNA using the PrimeScript RT reagent Kit with gDNA Eraser. qPCR was performed with TB Green premix Ex Taq II. β-actin was used as an internal control to calculate relative expression. PCR primer sequences are shown in Supplementary Tables S2, S3.

### Statistical analysis

The results were analyzed by GraphPad Prism 9.0; all data are expressed as means ± standard errors of the mean (SEM) and assessed for normality. Comparisons between two groups were assessed using unpaired Student’s t-tests; comparisons among three or more groups were conducted using one-way ANOVA followed by Turkey’s test. Differences with *p <* 0.05 were considered statistically significant.

## Results

### EMPA reduced body weight and fat mass, while improving lipid metabolism, in HFD mice

**FIGURE 1:**
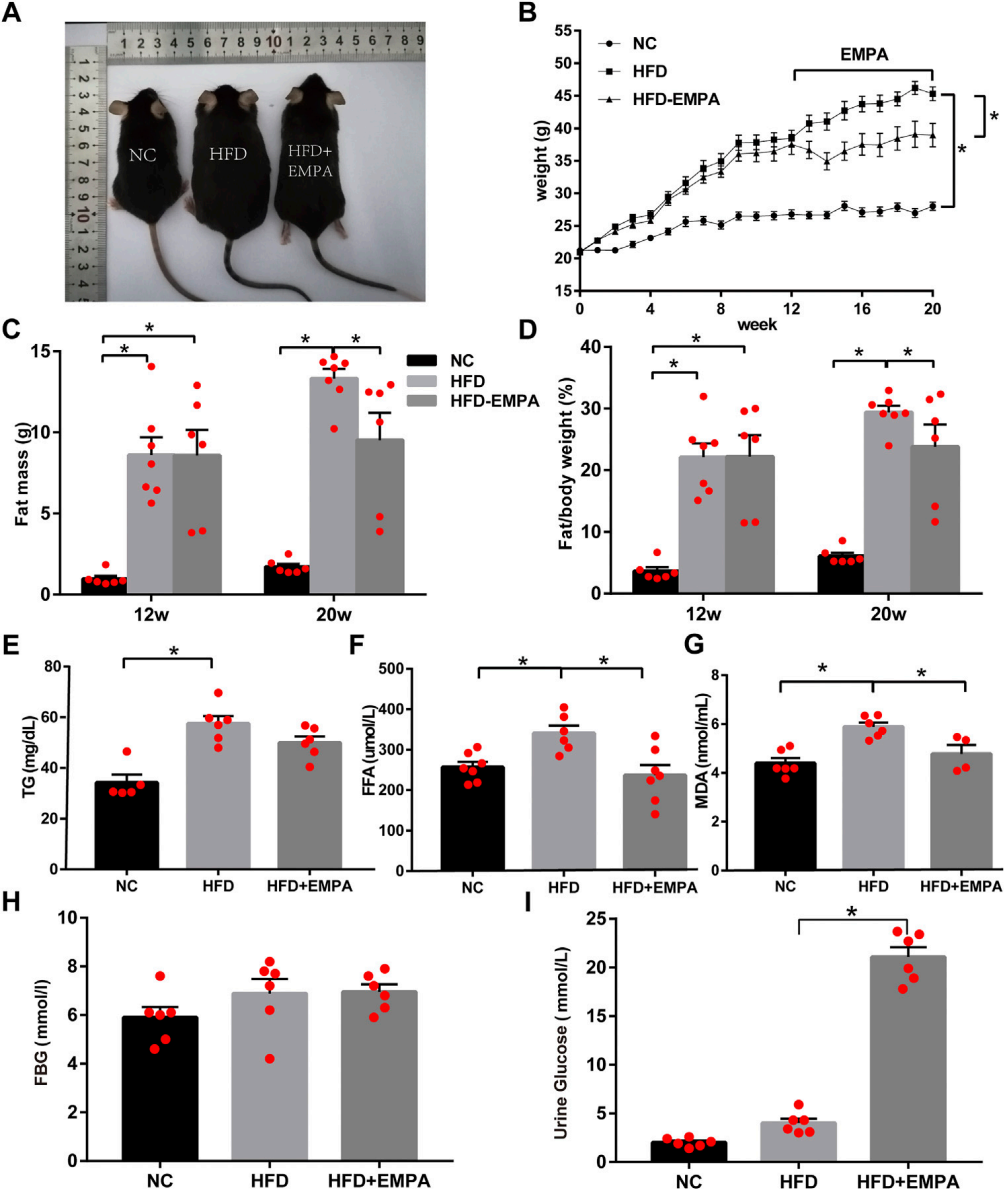
Body weight, fat mass and biochemical parameters in HFD mice with or without empagliflozin. **(A)** Morphology of mice. **(B)** Weekly body weights. **(C)** Body fat mass of the mice. **(D)** Body fat/body weight. **(E)** Plasma triglycerides levels. **(F)** Plasma free fatty acid levels. **(G)** Lipid peroxidation MDA levels. **(H)** Fasting glucose levels. **(I)** Urine glucose. Data are presented as means ± SEM (*n* = 4-6/group); *p < 0.05.

We established an HFD model in C57BL/6J mice. After 12 weeks, the HFD mice displayed significantly increased body weight and fat mass (*p* < 0.05). Eight-week administration of EMPA by oral gavage significantly suppressed both weight gain and fat mass (*p* < 0.05; Figures 1A–D). Additionally, circulating triglyceride, FFA, and lipid peroxidation (MDA) levels increased in HFD mice; these changes were alleviated after EMPA treatment (Figures 1E–G). Fasting blood glucose did not significantly differ among groups (*p* > 0.05; Figure 1H), but urine glucose was significantly increased after EMPA treatment (*p <* 0.05; Figure 1I). These results showed that EMPA alleviated metabolic alterations.

### EMPA improved hepatic function and alleviated hepatic lipid accumulation in HFD mice

**FIGURE 2:**
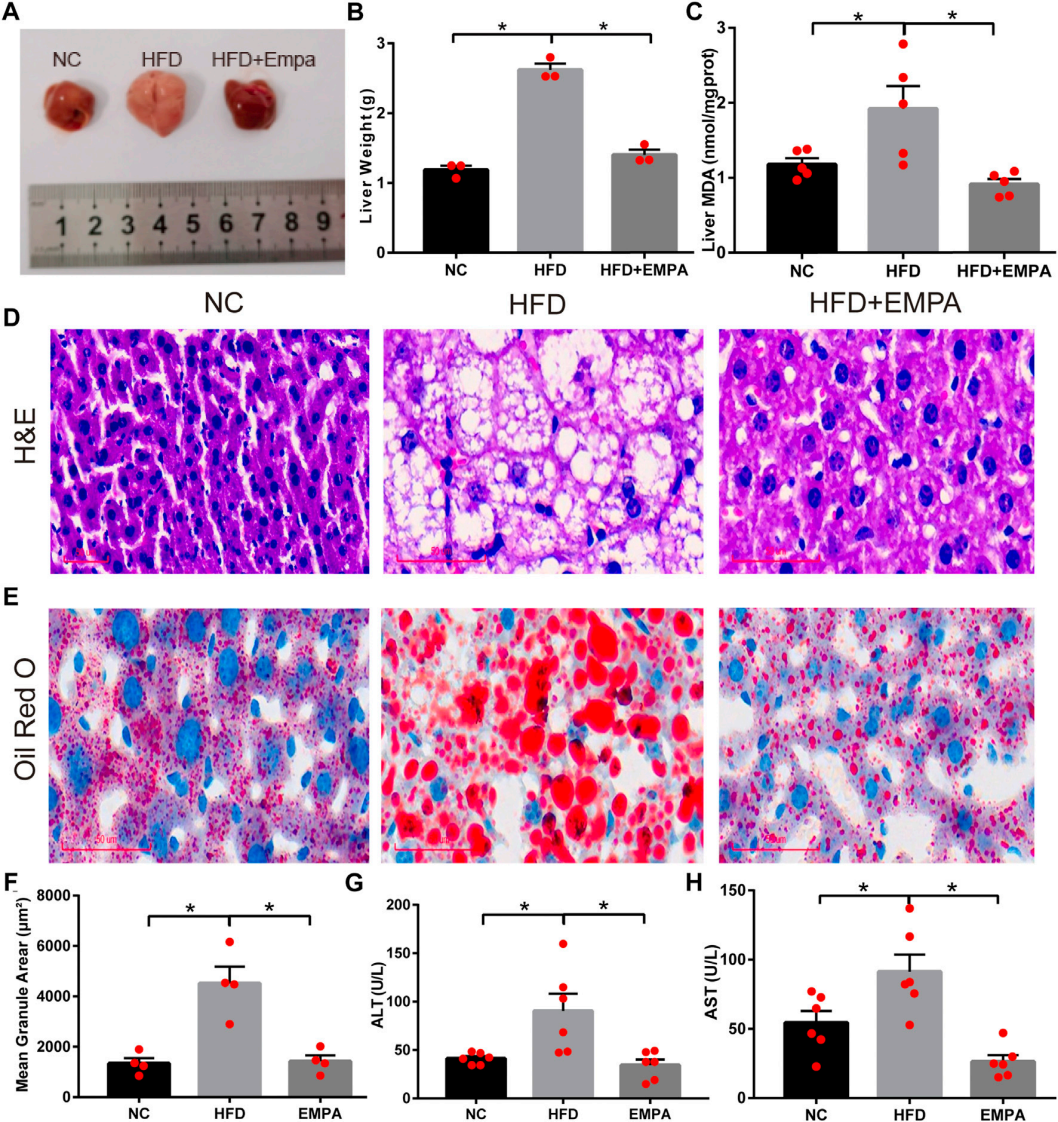
Empagliflozin alleviated hepatic function and lipid accumulation in HFD mice. **(A)** Liver morphology of mice. **(B)** Liver weight. **(C)** Liver MDA. **(D)** H&E staining. Scale bar, 50 μm. **(E)** Oil Red O staining. Scale bar, 50 μm. **(F)** quantification of Oil Red O. **(G)** Serum ALT levels. **(H)** Serum AST levels. Data are means ± SEM (*n* = 3–6/group). *p < 0.05.

Compared with control mice, HFD mice exhibited significantly greater liver mass, serum transaminase levels, and liver MDA content, indicating apparent hepatic injury after HFD induction (*p* < 0.05). EMPA treatment significantly reduced liver mass, liver MDA content, and serum transaminase levels, including both alanine aminotransferase and aspartate aminotransferase (*p* < 0.05; Figures 2A–H). Additionally, H&E and ORO staining showed noticeable histopathological changes, including increased hepatocyte volumes, more dispersed lipid vacuoles, and greater accumulation of lipid droplet deposition in HFD mice; these effects were alleviated by EMPA treatment (Figures 2D–F). These findings indicated that EMPA significantly reduced hepatic injury in HFD mice.

### EMPA upregulated Sesn2-mediated signaling in HFD mice

**FIGURE 3:**
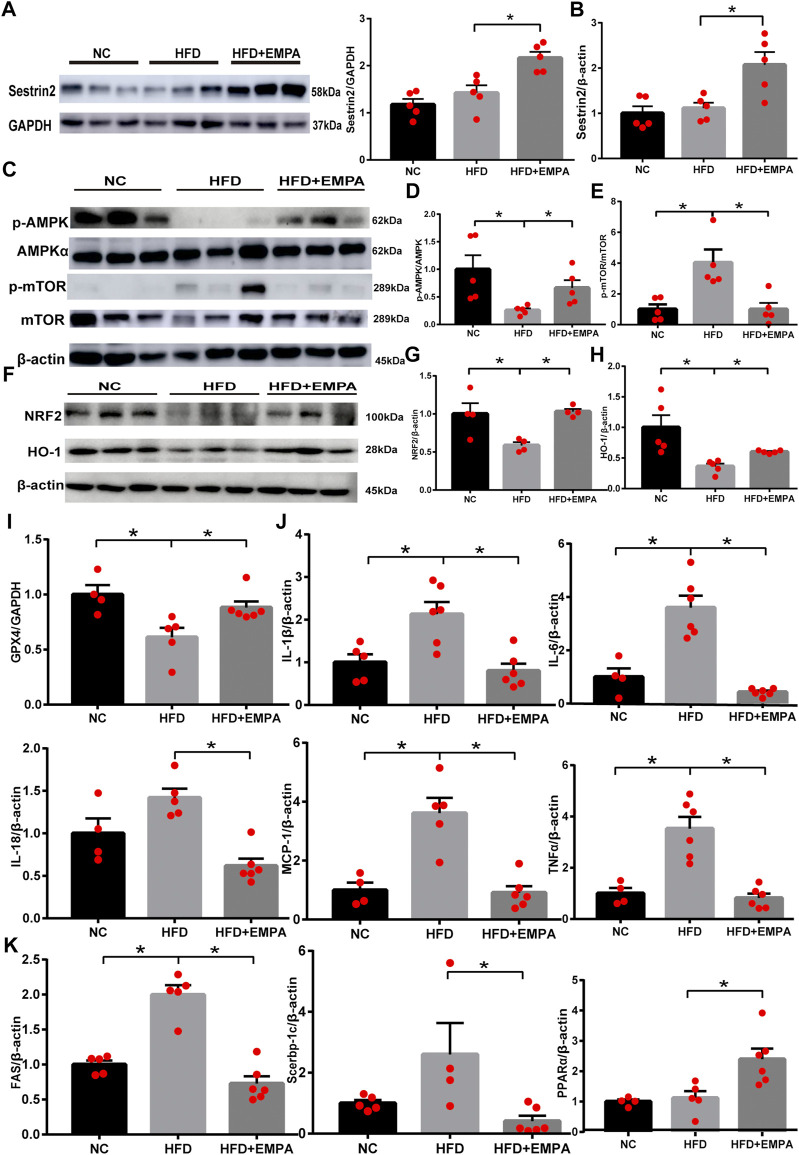
Empagliflozin upregulated the Sestrin2-mediated signaling pathway and protected HFD mice from hepatic inflammation and steatosis. **(A)** Western blotting analyses for Sestrin2. **(B)** mRNA expression of Sestrin2. **(C–H)** Western blotting analyses were conducted to detect the expression levels of p-AMPK, p-mTOR, and Nrf2/HO-1. **(I)** GPX4 mRNA. **(J)** mRNA expression of inflammatory cytokines. **(K)** qPCR results for genes associated with fatty acid synthesis and β-oxidation. Data are means ± SEM (n = 4-6/group). *p < 0.05.

To explore whether EMPA could activate Sesn2, we first evaluated the expression of liver Sesn2 via Western blotting. We found that HFD administration tended to enhance Sesn2 expression, but this difference was not statistically significant (*p* > 0.05); however, EMPA treatment upregulated Sesn2 expression (*p* < 0.05; Figure 3A). Consistent with the Western blotting results, RT-PCR analysis indicated that EMPA treatment upregulated Sesn2 mRNA expression (*p* < 0.05; Figure 3B). Because Sesn2 controls the AMPK-mTOR and oxidative stress pathways, we examined whether EMPA affected these pathways. EMPA treatment significantly increased AMPK phosphorylation and decreased mTOR phosphorylation (*p* < 0.05; Figures 3C–E). Additionally, HFD administration inhibited the expression of anti-oxidative stress pathway components Nrf2; the expression levels of Nrf2 and its target genes (GCLC and HMOX1) were enhanced after EMPA treatment (*p* < 0.05; Figures 3F–H, Supplementary Figure S1). Finally, HFD administration downregulated the lipid peroxide gene (*GPX4*), while EMPA treatment upregulated the expression of *GPX4* (*p* < 0.05; Figure 3I). These findings showed that EMPA upregulated the Sesn2-mediated signaling pathway and the anti-oxidative system to alleviate the onset of NAFLD.

### EMPA protected HFD mice from hepatic inflammation and steatosis

Next, we evaluated the effects of EMPA on NAFLD-related inflammation. Compared with control mice, HFD mice exhibited greater inflammation in the liver, as demonstrated by significant increases in the mRNA expression levels of *IL-6, IL-1β, IL-18, MCP-1*, and *TNF-α*. However, this HFD-related inflammation was suppressed by EMPA treatment (*p* < 0.05; Figure 3J). To more fully explore the molecular mechanism underlying the effects EMPA on lipid generation and metabolism, lipogenic and fatty acid β-oxidation genes were evaluated. As expected, HFD-induced disordered fatty acid oxidation in mice was significantly mitigated by EMPA treatment, as demonstrated by improvements in lipogenic and fatty acid β-oxidation genes (*FAS, Srebp-1c,* and *PPARα*) (*p* < 0.05; Figure 3K). These results illustrated that EMPA suppressed inflammation and steatosis during NAFLD development.

### EMPA activated Sesn2 and inhibited AMPK-mTOR signaling in PA-stimulated HepG2 cells

**FIGURE 4:**
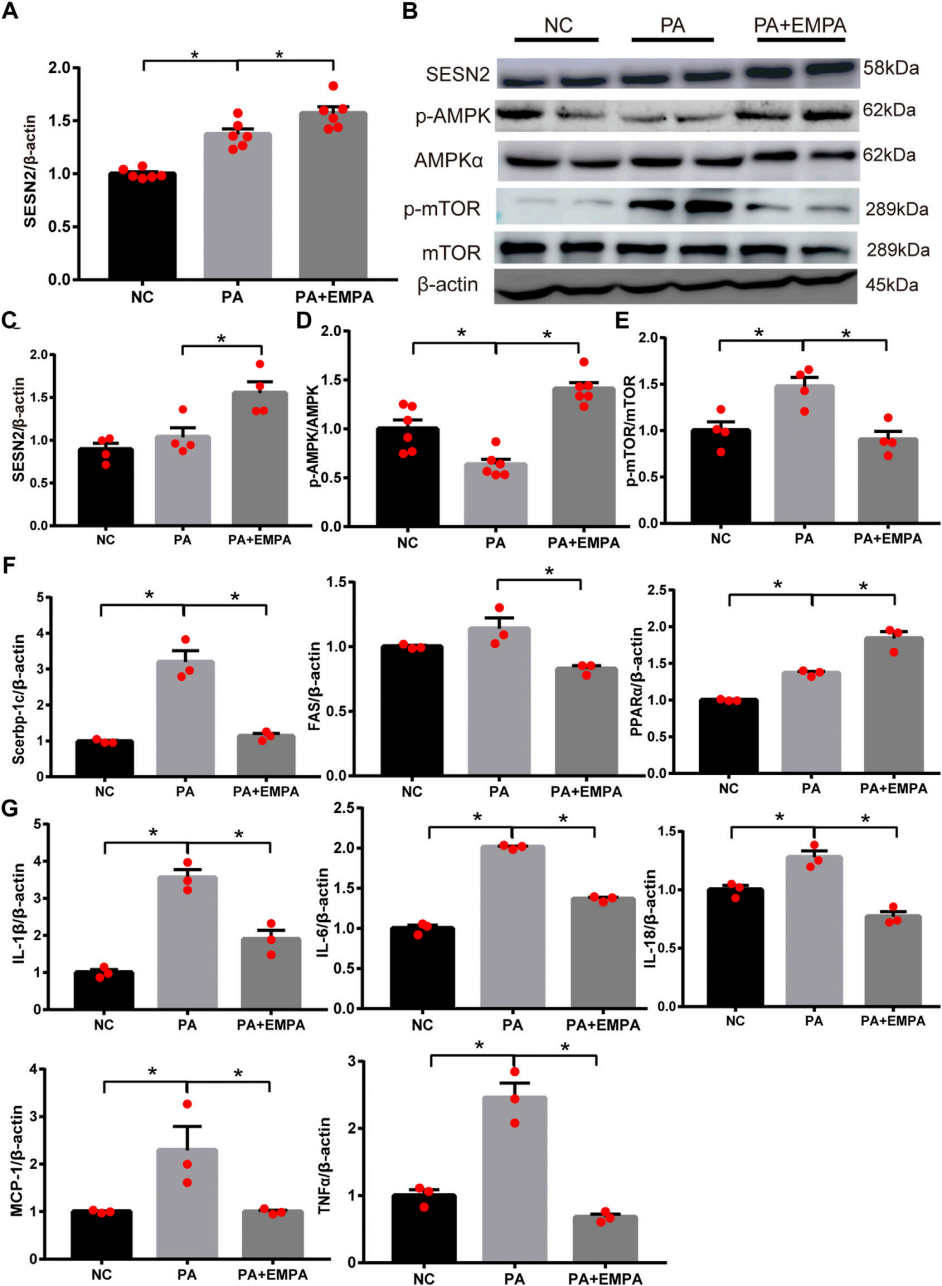
Effects of empagliflozin on palmitate-induced HepG2 cells. **(A)** Sestrin2 was quantified by qPCR. **(B–E)** Sestrin2, p-AMPK and p-mTOR protein levels. **(F)** The expressions of SREBP-1c, PPARα and fatty acid synthase (FAS) were quantified by qPCR. **(G)** Expression of inflammatory cytokines and chemokines in HepG2 cell. Data represent means ± SEM. *p < 0.05.

As expected, the relative mRNA expression and protein expression levels of Sesn2 were upregulated after treatment with EMPA (Figures 4A,B). Additionally, the protein expression levels of p-AMPK and p-mTOR were altered after EMPA treatment, similar to findings in the animal model (Figures 4B–E). To determine the molecular mechanism by which EMPA influences lipid generation, we evaluated the mRNA expression patterns of Srebp-1c, PPARα, and FAS transcriptional factors in HepG2 cells. These genes were increased in PA-stimulated cells, compared with control cells. However, these mRNA expression levels were significantly altered in EMPA-treated HepG2 cells (*p* < 0.05; Figure 4F). qPCR analysis indicated that mRNA expression levels of IL-18, IL-6, IL-1β, TNF-α and MCP-1 in HepG2 cells were significantly increased by PA stimulation; these mRNA expression levels were significantly downregulated after treatment with EMPA (*p* < 0.05; Figure 4G). Consistent with the *in vivo* results, our *in vitro* findings demonstrated the anti-inflammatory and anti-oxidative effects of EMPA against PA-stimulated HepG2 cells. These data showed that EMPA protected against fatty acid β-oxidation and inflammation; it also upregulated Sesn2 expression and activated the AMPK pathway in PA-stimulated HepG2 cells.

### EMPA activated the AMPK-mTOR signaling pathway by upregulating Sesn2 to protect against liver injury

**FIGURE 5:**
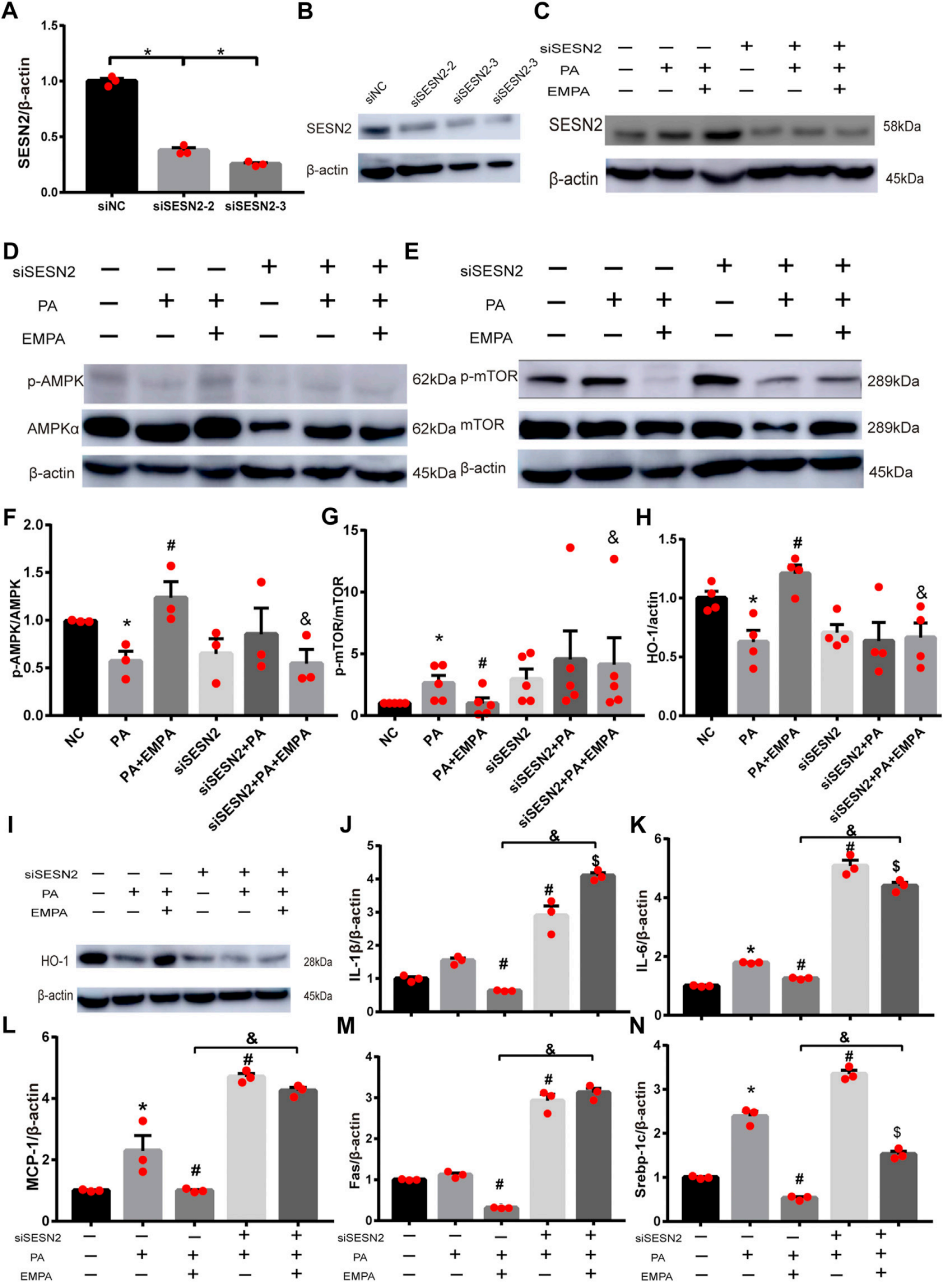
SESN2 knockdown weakened the effects of EMPA on inflammation and lipid accumulation. **(A)** The hepatocytes were transfected with siSESN2 for 48h, followed by qPCR analysis. **(B)** Western blot detection of SESN2 in HepG2 cells expressing SESN2-siRNA or control-siRNA. **(C)** Western blot analysis of SESN2 protein levels treated with or without EMPA after transfected with SESN2-siRNA or control-siRNA. **(D–I)** Western blot analysis of p-mTOR, p-AMPK and HO-1 protein levels after transfected with SESN2-siRNA or control-siRNA. **(J, K)** Inflammatory factors partly did not decrease in HepG2 cells with EMPA-siSESN2. **(L–N)** Fatty acid generation and β-oxidation in HepG2 cells treated with EMPA after SESN2-siRNA or control-siRNA. The data are expressed as the mean ± SEM; *p < 0.05 vs. siNC and #*p* < 0.05 . siNC + PA group; $*p* < 0.05 vs. siSESN2 002B; PA; &*p* < 0.05 vs. siNC + PA + EMPA group.

To explore whether Sesn2 was responsible for the beneficial effects of EMPA on hepatocytes, we transfected HepG2 cells with siRNA targeting Sesn2 (siSESN2) or with negative control siRNA (siNC). Transfection with siSESN2 downregulated Sesn2 expression in both PA-stimulated and PA-stimulated + EMPA-treated cells (Figures 5A–C). Additionally, EMPA did not activate p-AMPK or inhibit p-mTOR and HO-1, while it downregulated Sesn2 (Figures 5D–I); these findings indicated that EMPA activates AMPK via Sesn2. Notably, we did not observe decreased inflammatory factors in EMPA-treated HepG2 cells that had been subjected to Sesn2 knockdown (Figures 5J–L). Furthermore, Sesn2 downregulation led to enhanced lipid generation but not reduced fatty acid oxidation (PPARα) in PA-stimulated HepG2 cells; however, EMPA treatment partly reversed these changes (Figures 5M,N; Supplementary Figure S2). Taken together, our findings demonstrate that Sesn2 plays a critical role in inflammation and mediates the beneficial effect of EMPA on intracellular lipid metabolism in PA-stimulated cells.

### EMPA reduced lipid accumulation in PA-stimulated HepG2 cells

**FIGURE 6:**
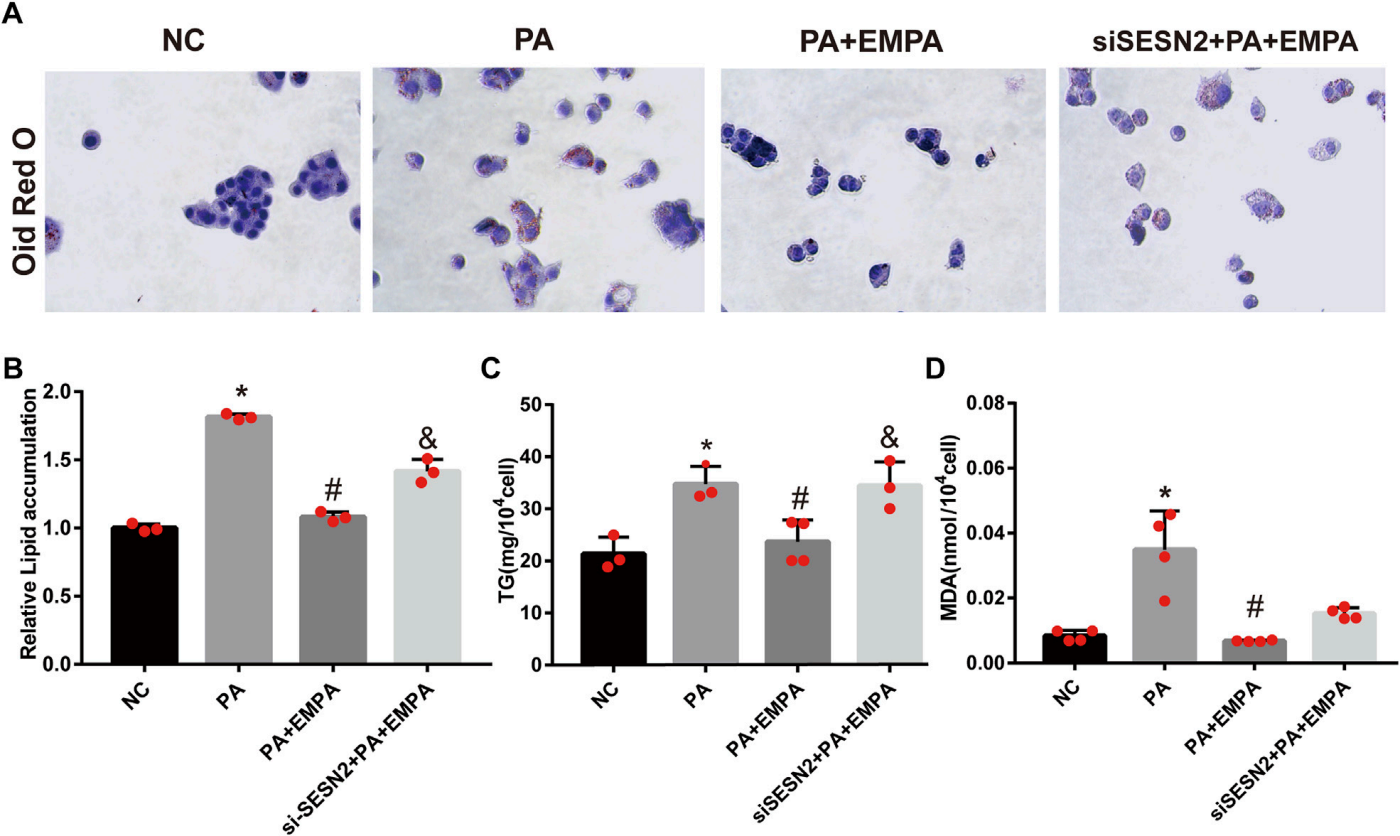
Empagliflozin reduces lipid accumulation in palmitate-induced HepG2 cells. **(A)** Lipid droplets in HepG2 cells were stained with Oil Red O. **(B)** Lipid accumulation was quantified by the absorbance value of the extracted Oil Red O dye at 495 nm. **(C)** TG in HepG2 cells. **(D)** MDA in HepG2 cells. Data are means ± SEM (*n* = 3-4/group). *p < 0.05 vs. NC and ^#^ *p* < 0.05 vs. PA group; ^&^ *p* < 0.05 vs. PA + EMPA group.

To confirm the beneficial effects of EMPA on lipid accumulation, we performed *in vitro* experiments using PA-stimulated HepG2 cells. After stimulation with PA, HepG2 cells were cultured either alone or in combination with EMPA for 24 h; lipid contents were examined by ORO staining. The results showed that lipid accumulation in HepG2 increased after stimulation with PA. However, the addition of EMPA led to significant reduction of lipid accumulation (Figures 6A–C). Moreover, to examine lipid peroxidation after PA stimulation in HepG2 cells, we measured the MDA content; EMPA significantly reduced MDA content in PA-stimulated cells (Figure 6D).

## Discussion

The present study demonstrated that EMPA treatment could attenuate HFD-induced NAFLD by activating Sesn2-mediated AMPK-mTOR signaling; this alleviated abnormal hepatic function and inflammation. Our results demonstrated the vital role for Sesn2 in EMPA-mediated changes in lipid metabolism and inflammation in models of NAFLD.

NAFLD has a complex pathogenesis. It is most prominently characterized by abnormal liver function and extensive lipid accumulation, particularly in patients with obesity (Saponaro et al., 2015). Excess FFA production causes disorders of lipid metabolism, lipid oxidation, and inflammation (Haukeland et al., 2006). In this study, HFD mice exhibited serum and hepatic lipid dysfunction, along with increases in body weight and fat mass. Moreover, HFD mice showed abnormal liver function with increased lipid drops in ORO staining and significant steatosis in H&E staining regarding the liver injury. These findings confirmed that HFD administration could induce NAFLD.

EMPA, an SGLT2i, has shown unprecedented benefits in clinical trials of patients with diabetes who have either established cardiovascular disease or NAFLD events (Cowie and Fisher, 2020; Sumida et al., 2020). Several pilot studies have shown that EMPA improves liver dysfunction or severe liver pathology through reductions of body weight, transaminase activity, fatty liver index, inflammatory response, and liver histopathology (steatosis or fibrosis) (Komiya et al., 2016; Seko et al., 2017; Sattar et al., 2018). Here, we found that EMPA improved hepatic function and alleviated hepatic lipid accumulation in HFD mice. The beneficial effects of EMPA might include reductions of inflammation and pro-inflammatory cytokine production, decreased lipid peroxidation, and increased energy utilization (Xu et al., 2017; Xu and Ota, 2018; Cowie and Fisher, 2020). However, the mechanism underlying these effects has not been fully elucidated. Our study confirmed that the NAFLD-related inflammation occurred in our *in vivo* model, as demonstrated by the significantly increased levels of IL-18, TNF-α, IL-6, MCP-1, and IL-1β; these levels were reduced after treatment with EMPA. This result is also confirmed by recent finding that EMPA treatments ameliorated hepatic pro-inflammatory cytokine genes (IL-1b, IL-6, and IFN-γ) and inflammatory chemokines (MCP-1, C-C motif chemokine ligand) in NAFLD mouse models (Lee et al., 2022). Consistent with the findings in previous studies, our *in vitro* experiments suggested that EMPA could reduce inflammation in PA-stimulated HepG2 cells.

Although inflammation is a major pathological component of NAFLD, the mechanism by which EMPA protects against NAFLD-related inflammation is not entirely clear. To explore this mechanism, we investigated the Sesn2-related signaling pathway. Sesn2 is a stress-responsive gene implicated in anti-oxidative processes. Several studies have demonstrated that Sesn2 controls physiological or pathophysiological processes through the AMPK/mTOR signaling pathway. Additionally, Sesn2 inhibits the accumulation of reactive oxygen species by activating Nrf2/HO-1 (Kim et al., 2017). Furthermore, Sesn2 reportedly rescued ischemic tolerance in aged hearts and promoted sensitivity to ischemic insults through the AMPK signaling pathway (Quan et al., 2018; Yan et al., 2021). The literature thus far suggests that Sesn2 is involved in controlling oxidative stress and hypoxia in multiple tissues (Sun X. et al., 2020). In our work, we found that EMPA upregulated Sesn2 to alleviate inflammation, while regulating downstream signals, including p-AMPK, p-mTOR, and Nrf2/HO-1. The *in vitro* findings showed that Sesn2 knockdown could aggravate the inflammatory response in PA-stimulated hepatocytes. Conversely, EMPA-mediated changes in inflammatory factors and AMPK/mTOR signaling were significantly inhibited by Sesn2 knockdown in PA-stimulated HepG2 cells. These findings suggested that EMPA-mediated effects on inflammation were partly dependent on Sesn2 expression.

EMPA has been reported to attenuate the accumulation of triglycerides and FFAs. The proposed mechanism depends partly on enhanced energy expenditure and increased fatty acid oxidation (Hawley et al., 2016; Xu et al., 2017). Lee *et al.* found that EMPA could alter the hepatic lipidome towards a protective profile (Lee et al., 2022). AMPK and mTOR complex (mTORC1 and mTORC2) primarily regulate cellular energy homeostasis (Garcia and Shaw, 2017; Liu and Sabatini, 2020). PA inhibits fatty acid oxidation via decreasing AMPKα Thr172 phosphorylation and its downstream target ACC phosphorylation (Mukhopadhyay et al., 2015). Hawley *et al.* showed that SGLT2i treatment activated AMPKα, thus accelerating fatty acid oxidation and decreasing liver lipid content (Hawley et al., 2016). Therefore, the AMPK pathway has been proposed to have a critical role in the EMPA-mediated enhancement of fatty acid oxidation. Additionally, circulating Sesn2 was independently associated with the level of high-density lipoprotein in a previous study, demonstrating that Sesn2 has a regulatory role in lipid metabolism (Bai et al., 2019). However, the mechanisms underlying the effects of Sesn2 on lipid metabolism and fatty acid oxidation have been unclear.

Lipid metabolism contains the fatty acids and triglycerides metabolism, involving in triglycerides accumulation, fatty acid oxidation, and *de novo* lipogenesis (Jeon and Carr, 2020). Here, we observed that EMPA enhanced fatty acid oxidation, as demonstrated by the significantly increased levels of PPARα and GPX4. PPARα, as a critical transcriptional regulator, mainly participates in mitochondrial oxidation and whose ligands include FFAs and fatty acid derivatives (Mandard et al., 2004). GPX4, a lipid peroxide regulator, can protect cells from death by transforming lipid hydroperoxides into lipid alcohol (Forcina and Dixon, 2019). Our study showed that EMPA regulated lipid oxidation by enhancing GPX4 and PPARα. Moreover, we explored the related oxidation pathway such as Nrf2/HO-1pathway. After Nrf2 enters the nucleus, its downstream targets, including HO-1(HMOX1) and GCLC, appear to be induced (Koyani et al., 2016). In our study, we accessed the lipid oxidation pathway; the results indicated that EMPA activated the Nrf2/HO-1/GCLC axis to reduce oxidation. However, the mechanisms underlying the effects of Sesn2 on lipid and fatty acid oxidation have been unclear. Although we identified that EMPA ameliorates lipid oxidation via Sesn2 knockdown, PPARα was not inhibited after treatment with EMPA. This suggested that Sesn2 did not improve lipid oxidation by regulating PPARα.

Additionally, we observed that EMPA reduced lipid metabolism, as demonstrated by the significantly reduced levels of FAS and Srebp-1c and decreased TG and FFA levels. FAS and Srebp-1c are the essential genes involved in *de novo* lipogenesis (Obara et al., 2010). Furthermore, silencing of Sesn2 caused upregulation of lipogenic processes and increased TG and FFA levels. After treatment with EMPA, the FAS and Srebp-1c levels were significantly decreased but remained higher than in cells with unaltered Sesn2 expression. Meanwhile, EMPA could not reduce lipid accumulation (ORO), TG and FFA levels in HepG2 cells after silencing Sesn2. These findings suggested that EMPA ameliorates changes in lipid metabolism partly through downregulation of lipogenic processes via upregulation of Sesn2.

This study had a few limitations. First, we did not perform experiments in Sesn2 knock-out mice. Second, we did not fully explore how EMPA activates Sesn2. Third, this study does not determine whether EMPA alleviated lipid metabolism and synthesis via Sesn2 or whether PPARα/AMPK is dependent on these alterations. Finally, although we explored p-AMPK (Thr172) and p-mTOR (mTORC1), the downstream targets acetyl-CoA carboxylase and mTORC2 were not identified. Therefore, further studies are needed to elucidate the mechanism and explore the effects of Sesn2 silencing in an *in vivo* model.

In summary, our results showed that EMPA activates Sestrin2-mediated AMPK/mTOR pathway and ameliorates lipid accumulation in obesity-related nonalcoholic fatty liver disease. Thus, we propose Sesn2 as a target for treatment of NAFLD through its effects on the AMPK/mTOR signaling pathway.

## Data Availability

The original contributions presented in the study are included in the article/Supplementary Material, further inquiries can be directed to the corresponding authors.
