# Supplementary material for: Empagliflozin activates Sestrin2-mediated AMPK/mTOR pathway and ameliorates lipid accumulation in obesity-related nonalcoholic fatty liver disease
Source: Front Pharmacol. 2022 Sep 5;13:944886. doi: 10.3389/fphar.2022.944886 (PMC9483033; doi:10.3389/fphar.2022.944886)
Supplement: Supplementary file 1 [file DataSheet1.docx]

**Empagliflozin Upregulates Sestrin2 to Ameliorate Inflammation via AMPK/mTOR-dependent Effects in Non-alcoholic Fatty Liver Disease**

**Supplemental Materials**

**Supplementary table 1**

| **Reagent** | **Company** |
| --- | --- |
| Whole Cell Lysis Assay | KeyGEN (Nanjing, China) |
| TRIZOL | Thermo Fisher Scientific (Waltham, USA) |
| Aspartate Aminotransferase (AST) Assay Kit | Jiancheng (Nanjing, China) |
| Alanine Aminotransferase (ALT) Assay Kit | Jiancheng (Nanjing, China) |
| Triglyceride (TG) Assay Kit | Jiancheng (Nanjing, China) |
| Lipid peroxidation MDA Assay Kit | Jiancheng (Nanjing, China) |
| FFA assay kit | Solarbio (Beijing, China) |
| BCA Protein Kit | Solarbio (Beijing, China) |
| Antibodies of p-AMPK, AMPK, β-actin, mTOR, p-mTOR, Nrf2 and HO-1 | CST (Danvers, USA) |
| Antibodies of PGC1α and Sestrin2 | Proteintech(Wuhan, China). |
| Mouse-Body Composition Analyzer | Bruker，Germany |
| HepG2 Cell | Type Culture Collection of the Chinese Academy of Sciences (Shanghai, China) |
| Fetal Bovine Serum | Gibco (Thermo Fisher Scientific, China) |
| Palmitic Acid | Kunchuang(Xi'an, China) |
| ORO Stain Kit | Solarbio (Beijing, China) |
| Lipofectamine 3000 | Invitrogen, USA |

**Supplementary table 2. Primer sequences of mouse for RT-qPCR analysis.**

| **Gene** | **Sequence (5'-3')** |
| --- | --- |
| *Sestrin2* (mouse) | Forward: TCCGAGTGCCATTCCGAGAT |
|  | Reverse: TCCGGGTGTAGACCCATCAC |
| *Fas* (mouse) | Forward: TATCAAGGAGGCCCATTTTGC |
|  | Reverse: TGTTTCCACTTCTAAACCATGCT |
| *PPARα* (mouse) | Forward: AGAGCCCCATCTGTCCTCTC |
|  | Reverse: ACTGGTAGTCTGCAAAACCAA |
| *Srebp-1*c (mouse) | Forward: GGAGCCATGGATTGCACATT |
|  | Reverse: GGCCCGGGAAGTCACTGT |
| *GPX4* (mouse) | Forward: GCAACCAGTTTGGGAGGCAGGAG |
|  | Reverse: CCTCCATGGGACCATAGCGCTTC |
| *MCP-1* (mouse) | Forward: TAAAAACCTGGATCGGAACCAAA |
|  | Reverse: GCATTAGCTTCAGATTTACGGGT |
| *TNF-α* (mouse) | Forward: CCTGTAGCCCACGTCGTAG |
|  | Reverse: GGGAGTAGACAAGGTACAACCC |
| *IL-18* (mouse) | Forward: GACTCTTGCGTCAACTTCAAGG |
|  | Reverse: CAGGCTGTCTTTTGTCAACGA |
| *IL-1β* (mouse) | Forward: GCAACTGTTCCTGAACTCAACT |
|  | Reverse: ATCTTTTGGGGTCCGTCAACT |
| *GCLC* (mouse) | Forward: CTACCACGCAGTCAAGGACC |
|  | Reverse: CCTCCATTCAGTAACAACTGGAC |
| *HMOX1* (mouse) | Forward: AGGTACACATCCAAGCCGAGA |
|  | Reverse: CATCACCAGCTTAAAGCCTTCT |
| *β-actin*(mouse) | Forward: GGCTGTATTCCCCTCCATCG |
|  | Reverse: CCAGTTGGTAACAATGCCATGT |

**Supplementary table 3. Primer sequences of human for RT-qPCR analysis.**

| **Gene** | **Sequence (5'-3')** |
| --- | --- |
| *SESN2*(human) | Forward: TCTTACCTGGTAGGCTCCCAC |
|  | Reverse: AGCAACTTGTTGATCTCGCTG |
| *Fas*(Human) | Forward: AGATTGTGTGATGAAGGACATGG |
|  | Reverse: TGTTGCTGGTGAGTGTGCATT |
| *Srebp-1c*(Human) | Forward: GCCCCTGTAACGACCACTG |
|  | Reverse: CAGCGAGTCTGCCTTGATG |
| *PPARα*(human) | Forward: CGGTGACTTATCCTGTGGTCC |
|  | Reverse: CCGCAGATTCTACATTCGATGTT |
| *IL-1β*(human) | Forward: TTCGACACATGGGATAACGAGG |
|  | Reverse: TTTTTGCTGTGAGTCCCGGAG |
| *IL-6*(human) | Forward: CTGCAAGAGACTTCCATCCAG |
|  | Reverse: AGTGGTATAGACAGGTCTGTTGG |
| *IL-18*(human) | Forward: TCTTCATTGACCAAGGAAATCGG |
|  | Reverse: TCCGGGGTGCATTATCTCTAC |
| *TNF-α*(human) | Forward: GAGGCCAAGCCCTGGTATG |
|  | Reverse: CGGGCCGATTGATCTCAGC |
| *MCP-1*(human) | Forward: ACCAGCAGCAAGTGTCCCAAAG |
|  | Reverse: TTTGCTTGTCCAGGTGGTCCATG |
| *GPX4*(human) | Forward: GAGGCAAGACCGAAGTAAACTAC |
|  | Reverse: CCGAACTGGTTACACGGGAA |
| *β-actin*(human) | Forward: CATGTACGTTGCTATCCAGGC |
| *siSesn2*(human) | sense: CCGAAGAAUGUACAACCUCCU |
|  | antisense: AAGAGGUUGUACAUUCUUCGG |


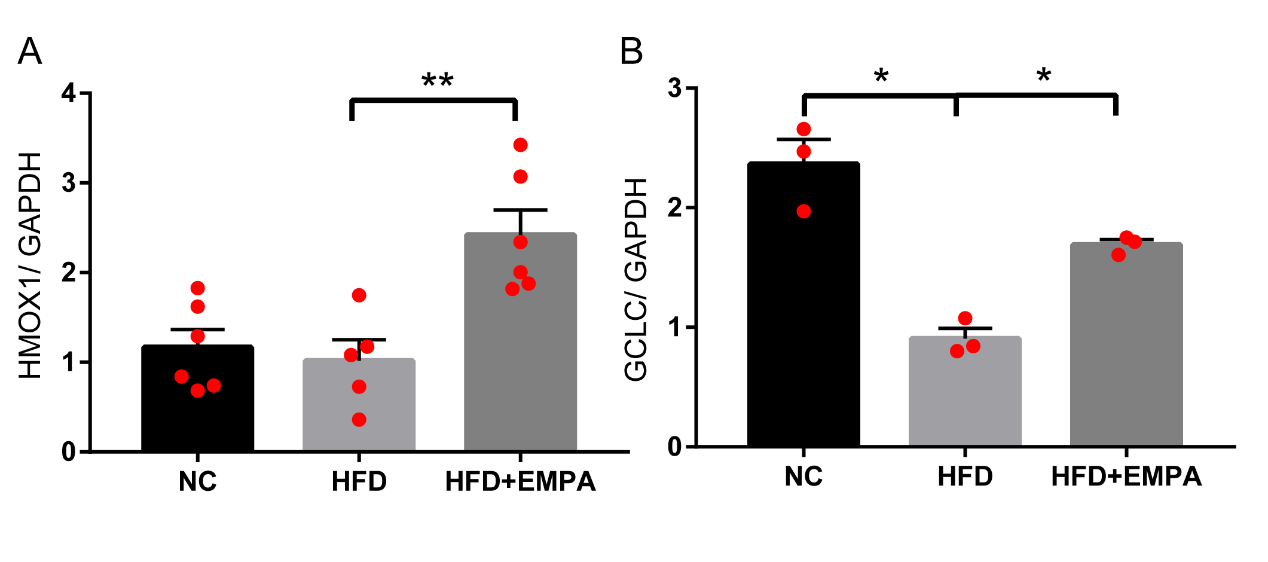


**Supplemental Figure 1.** Effects of EMPA treatment on HMOX1 (A) and GCLC (B).

n=3-6/group. **p*<0.05.


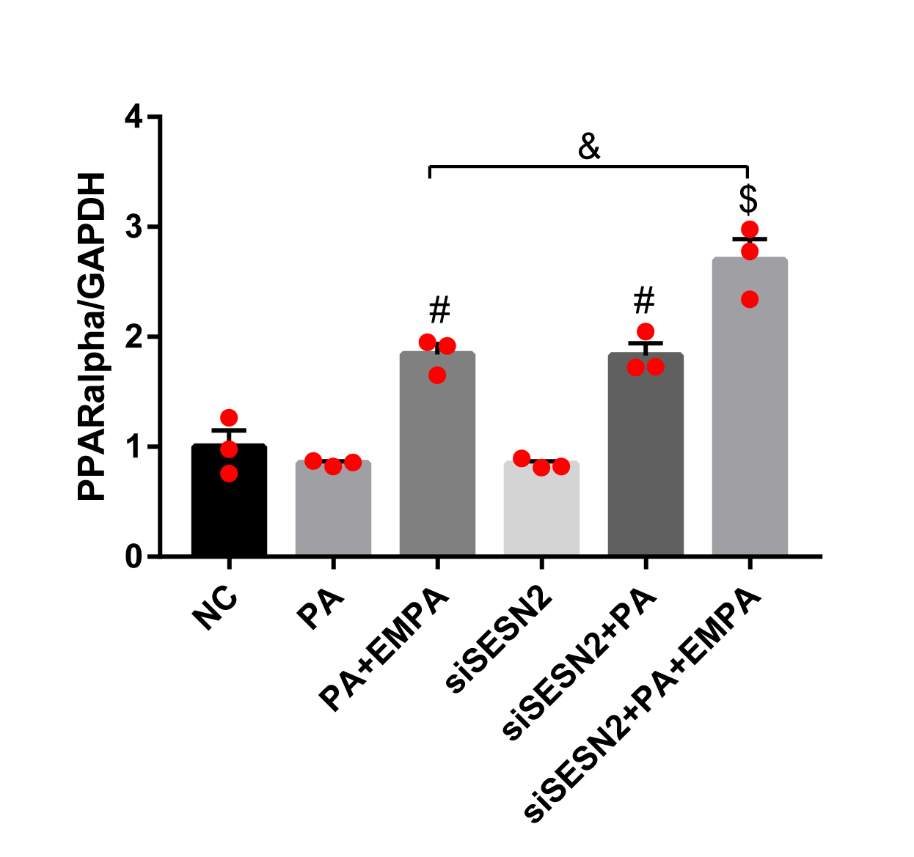


**Supplemental Figure 2.** Effects of EMPA treatment on PPARα.

The data are expressed as the mean ± SEM; * *p* <0.05 vs. siNC and # *p* < 0.05 vs. siNC+ PA group; $ *p* < 0.05 vs. siSESN2 + PA; & *p* < 0.05 vs. siNC + PA + EMPA group.
